# Supplementary material for: Physical measures of physical functioning as prognostic factors to predict outcomes in low back pain: Protocol for a systematic review
Source: PLoS One. 2023 Dec 11;18(12):e0295761. doi: 10.1371/journal.pone.0295761 (PMC10712879; doi:10.1371/journal.pone.0295761)
Supplement: S1 File — This file shows MEDLINE search strategy. (PDF) [file pone.0295761.s002.pdf]

Database(s): **Ovid MEDLINE(R) ALL** 1946 to June 30, 2023
Search Strategy:

#	Searches
1	Low Back Pain/
2	(low* adj5 back adj5 (pain* or ache*)).tw,kf.
3	(backache* adj4 low*).tw,kf.
4	(backpain* adj4 low*).tw,kf.
5	(lumbar or lumbosacral or lumbo-sacral or low* back*).tw,kf.
6	(lumbago or sciatica).tw,kf.
7	(radiculopathy or radiculitis or radicular pain*).tw,kf.
8	1 or 2 or 3 or 4 or 5 or 6 or 7
9	(physical outcome measur* or physical measures of function*or measures of function* or physical function* test).tw,kf.
10	(range of motion or ROM).tw,kf.
11	Schober.tw,kf.
12	(finger* adj3 (floor or toe or knee or fibular head)).tw,kf.
13	(wrist crease adj3 floor).tw,kf.
14	(straight leg raise* or SLR or Lasegue*).tw,kf.
15	(isometric strength or isokinetic strength).tw,kf.
16	(motor control or movement control or functional movement screen* or FMS or sitting one leg knee extension or posterior pelvic tilt or waiter's bow or one leg stance).tw,kf.
17	gait/ or gait analysis/
18	walking speed/
19	(spatiotemporal gait or spatio temporal gait or stride length or stride duration or gait speed or cadence or gait asymmetry or stance phase or swing phase or double limb support or single limb support).tw,kf.
20	(inclinometer or goniometer or kyphometer or electromagnetic tracking).tw,kf.
21	muscle strength dynamometer/
22	(dynamometer or manual muscle test or MedX or Cybex or Kin-Com or RehaGait or JAMAR).tw,kf.
23	(aerobic capacity or VO2* or bicycle ergomet* or maximal graded exercise* or steep ramp).tw,kf.

24	(Biering Sorensen or modified Sorensen).tw,kf.
25	Roman chair.tw,kf.
26	(sternum adj3 (ground or floor)).tw,kf.
27	muscle endurance.tw,kf.
28	((back or body or trunk) adj3 endurance).tw,kf.
29	prone bridge.tw,kf.
30	((lower extremities or (hips and knees)) adj5 (90deg* or "90 degree*")).tw,kf.
31	(arch-up* or sit-up* or squat* or dumbbell press*).tw,kf.
32	(double limb stance or single limb stance or stork stand* or flamingo balance or y-balance).tw,kf.
33	(clinical test* adj2 sensory interaction adj2 balance).tw,kf.
34	(Berg balance scale or Tinetti* or performance oriented mobility assessment* or tandem walk*).tw,kf.
35	lower extremity motor coordination test*.tw,kf.
36	(chair adj3 (stand* or rise*)).tw,kf.
37	(sit to stand or stand up or stand ups or roll*).tw,kf.
38	(lie adj2 sit).tw,kf.
39	(bed adj2 chair).tw,kf.
40	step*.tw,kf.
41	lifting/
42	(lift* or progressive isoinertial lifting evaluation or pile).tw,kf.
43	(forward reach or functional reach).tw,kf.
44	walk test/
45	(self-paced walk* or 4-meter walk* or 4-metre walk* or 5-meter walk* or 5-metre walk* or 10-meter walk* or 10-metre walk* or 15-meter walk* or 15-metre walk* or 50-meter walk* or 50-metre walk* or 50-foot walk* or 5-minute walk* or 6-minute walk* or treadmill or overground walk*).tw,kf.
46	(walk adj3 hall*).tw,kf.
47	shuttle walk*.tw,kf.
48	(stair* adj2 climb*).tw,kf.
49	("timed up and go" or TUG or "8 foot up and go").tw,kf.
50	accelerometry/
51	wearable electronic devices/ or fitness trackers/
52	(acceleromet* or activity monitor or pedomet* or GPS or watch or smartwatch).tw,kf.
53	(6WT adj3 app*).tw,kf.
54	functional capacity evaluation.tw,kf.

55	9 or 10 or 11 or 12 or 13 or 14 or 15 or 16 or 17 or 18 or 19 or 20 or 21 or 22 or 23 or 24 or 25 or 26 or 27 or 28 or 29 or 30 or 31 or 32 or 33 or 34 or 35 or 36 or 37 or 38 or 39 or 40 or 41 or 42 or 43 or 44 or 45 or 46 or 47 or 48 or 49 or 50 or 51 or 52 or 53 or 54
56	exp Risk/
57	risk.tw.
58	exp Cohort Studies/
59	cohort.tw.
60	exp Prognosis/
61	"prognos*".tw.
62	"predict*".tw.
63	exp Incidence/
64	incidence.tw.
65	exp Survival Analysis/
66	survival.tw.
67	"causal factor".tw.
68	course.tw.
69	56 or 57 or 58 or 59 or 60 or 61 or 62 or 63 or 64 or 65 or 66 or 67 or 68
70	8 and 55 and 69
