# Supplementary material for: Physical measures of physical functioning as prognostic factors to predict outcomes in low back pain: Protocol for a systematic review
Source: PLoS One. 2023 Dec 11;18(12):e0295761. doi: 10.1371/journal.pone.0295761 (PMC10712879; doi:10.1371/journal.pone.0295761)
Supplement: Attachment — Submitted filename: Response to Reviewers - Rameeza Rashed.pdf [file pone.0295761.s004.pdf]

Date: 22-Aug-2023

Subject: Appeal

Dear Academic Editor,

Thank you for your review and consideration of our manuscript titled "Physical measures of physical functioning as prognostic factors to predict outcomes in low back pain: protocol for a systematic review" (PONE-D-23-23995) for publication in PLOS ONE.

We have carefully reviewed your comments and we are respectfully requesting a review of your decision to reject the manuscript. We believe the points you have raised about the clarity and omissions in the design of this systematic review protocol, written English not being of an academic standard, and the value of the systematic review topic being questionable are not accurate and are comprehensively addressed within the submitted manuscript. We address each point in turn for your clarity.

Clarity and omissions in the design of the systematic review protocol

There were several comments indicating the systematic review protocol methodology was unclear, with suggestions for alternative section headings to improve clarity and comprehensiveness. The headings we have used within the submitted systematic review protocol manuscript align exactly with the required headings in the gold standard for reporting of systematic review protocols, the Preferred Reporting Items for Systematic Reviews and Meta-Analyses Protocols (PRISMA-P) statement.

There were also suggestions to change the structure of eligibility criteria to enhance clarity. The structure of eligibility criteria we have used aligns with the Cochrane Prognosis Methods Group, which has established best-evidence guidelines for developing protocols of systematic reviews of prognostic studies such as this.

There were also several comments indicating that some elements of design were omitted. For example, the comments indicate the date of searching each database is missing. However, the search dates of all electronic databases are clearly detailed in the manuscript, from inception to June 30, 2023 in MEDLINE, EMBASE, CINAHL, and Scopus. We believe all required systematic review protocol methodological design components are clearly and comprehensively addressed, which is supported by the completed PRISMA-P checklist submitted with the manuscript.

Written English is not an academic standard

Considerable care was taken to ensure that the manuscript adheres to established academic language conventions. We have, with due diligence, followed recognized guidelines for scientific writing, including proper grammar, syntax, accurate terminology, and writing conventions in the PLOS ONE author guidelines. Prior to submission, the manuscript was reviewed numerous times by the supervisory team which consists of three English as a first language speakers. We are confident we can fully address any specific feedback to enhance the clarity of writing.

Value of this topic

The value of the topic of this systematic review protocol is questioned. The introduction to the protocol provides a strong rationale for why this systematic review is required and highlights the value of this topic. For example, knowledge of prognostic factors of outcome for low back pain offers a proactive and personalized approach to patient care. Physical measures are an emerging area of research that offers promise for a comprehensive assessment of low back pain to enable improved patient outcomes. Synthesizing the literature on physical measure prognostic factors to predict outcomes in low back pain will provide essential knowledge to aid identification of high-risk patients for targeted allocation of health care resources and support for improved patient outcomes.

The justification for the importance, need and value of this topic may have been missed as the comments indicate the introduction was not read. Further, the comments indicate the results, discussion and conclusions are questionable. However, as this is a protocol for a systematic review, there are no results or conclusions at this stage of research. We respectfully suggest that this misunderstanding may have contributed to the interpretation of questionable value of our manuscript.

We are confident that this systematic review protocol is a high-value topic that will be of interest to PLOS ONE readers.

<u>In summary</u>

Based on our evaluation of the 3 reasons highlighted above for rejection of this manuscript, we kindly request a reconsideration of the reject decision on this systematic review protocol. We would like to emphasize that our manuscript underwent rigorous internal review and revision to ensure that it adheres to the high standards of PLOS ONE in advance of submission. Our team remains committed to delivering quality research that contributes to the academic community and advances the field. We feel strongly this manuscript will be a valuable publication for PLOS ONE given its methodological rigor and value.

If necessary, we are more than willing to work closely with the reviewers and the editorial team to further clarify any aspects of the manuscript that may have been misunderstood or misinterpreted. Our aim is to ensure that our research is accurately assessed based on its scientific merit and contribution.

Thank you for your time and consideration. We eagerly await your response and hope for a positive outcome.

Sincerely,

Rameeza Rashed, on behalf of all authors

rrashed2@uwo.ca
