# Supplementary material for: Physical measures of physical functioning as prognostic factors to predict outcomes in low back pain: Protocol for a systematic review
Source: PLoS One. 2023 Dec 11;18(12):e0295761. doi: 10.1371/journal.pone.0295761 (PMC10712879; doi:10.1371/journal.pone.0295761)
Supplement: Attachment — Submitted filename: 1_Comment for LBP_protocol for review.doc [file pone.0295761.s003.doc]

**Comments**

**General comments**

As there are a number of omissions in the design of this study and written English is not academic standard, the value of this topic under review is questionable.

**Abstract/ Introduction-content**

No read

**Methods: General**

-It would be better to have two subtitles as “Identification and selection of studies” and “Assessment of characteristics of studies”.

-Information is messy, so it makes readers feel difficult to read.

**Methods: Identification and selection of studies**

Information is messy. Inclusion criteria should be following as Design (missing what kind of design would be included), Participants (need to defined the low back pain), Outcome measures (no enough information)

***Searches***

1. For each database, it should be as example like: Embase (1947 to July 2023). This is missing

***Inclusion criteria: this should be part of Identification and selection of studies***

Authors should tidy up information, perhaps using a table to cover all area, such as Design, Participants, Intervention, Outcome measures, Comparisons.

**Methods: Assessment of characteristics of studies**

There should be more points in this part. It would be better to have this headings and follow some subheading as below:

*Quality:* missing

*Participants:* missing

*Intervention:* messing

*Outcome measures:* missing

**Results/ Discussion/ Conclusion: context.**

As there are so many questions in the method, results, discussion and conclusion are questionable.

**Readability and style.**

This paper is not easy to read, because the structure of written information.
